# Trustworthy AI-Augmented Objective Structured Clinical Examinations in Nursing Education: Taiwan-Japan Viewpoint on 5 AI Roles, Governance, and Cross-Border Implementation

**DOI:** 10.2196/87830

**Published:** 2026-03-12

**Authors:** Kazumi Kubota, Ayako Nishimura, Ryoma Seto, Liu Li

**Affiliations:** 1 Research Organization Shimonoseki City University Shimonoseki, Yamaguchi Japan; 2 Department of Healthcare Information Management University of Tokyo Hospital Tokyo Japan; 3 Faculty of Healthcare, Division of Nursing Tokyo Healthcare University Tokyo Japan; 4 Faculty of Healthcare, Division of Healthcare Informatics Tokyo Healthcare University Tokyo Japan; 5 Show Chwan Health Care System Taipei Taiwan

**Keywords:** objective structured clinical examination, OSCE, nursing education, generative artificial intelligence, generative AI, standardized patient, assessment governance, transparency and traceability, Taiwan-Japan collaboration, artificial intelligence, AI

## Abstract

Generative artificial intelligence (AI) is arriving in high-stakes assessment; however, governance, validity evidence, and faculty readiness remain uneven. From a Taiwan-Japan perspective, we outline a pragmatic, transferable approach to integrating AI into nursing objective structured clinical examinations (OSCEs) using a 5-AI-role model—learning assistant, AI‑augmented standardized patient, assessment assistant, case generator, and learning analyst—mapped across pre-OSCE, peri-OSCE, and post-OSCE workflows with human-in-the-loop final judgment. Taiwan contributes agile interdisciplinary development, staged pilots (practice, mock OSCE, and limited high-stakes stations), A/B comparisons, and explainability-by-design logging that links scores to time-stamped evidence. Japan contributes robust policy scaffolding (national AI use guidance in K-12, a revised nursing model core curriculum with outcomes and assessment blueprints, and institutional research cultures that support auditability and quality assurance). We distill 4 cross-cutting governance pillars—human oversight, learning process transparency, ethics and safety, and traceability—into implementable techniques (machine-readable rubrics, standardized patient persona cards, bias monitoring, and targeted faculty development). Aligning with international principles (International Advisory Committee for AI; Organisation for Economic Co-operation and Development; United Nations Educational, Scientific and Cultural Organization; World Health Organization; European Commission’s High Level Expert Group; and National Institute of Standards and Technology), we propose a joint road map and shared registry to benchmark reliability, validity, equity, and workload impact. This viewpoint targets OSCE directors, nursing educators, and institutional leaders and provides a phase-gated governance blueprint rather than reporting original trial outcomes. Taiwan-led agility, complemented by Japan’s standards-driven assurance, can form an Asia-Pacific reference model for trustworthy AI‑augmented OSCE in nursing education.

## Introduction

Generative artificial intelligence (AI) is steadily making its way into health professions education, but its use in high-stakes assessment remains measured—and rightly so. Benefits will be realized only if reliability, validity, fairness, and accountability are protected and can be shown in the day‑to‑day reality of teaching and examining [1-6]. Objective structured clinical examinations (OSCEs) have long provided a structured approach to eliciting clinical performance, and the method has matured through improvements in station design, rater training, and standard setting [7,8]. Contemporary assessment programs also push beyond single-event decisions toward programmatic assessment, in which evidence accumulates over time and feedback is central to learning [9]. Therefore, validity arguments must connect the blueprint and rubric to what we actually observe and to the decisions we ultimately make [10,11]. Against this backdrop, the question for many nursing programs is not whether AI will touch OSCEs but how to make it help rather than harm.

This viewpoint takes a pragmatic stance from a Taiwan-Japan collaboration. Taiwan brings agile interdisciplinary development, with short iteration cycles and pilots instrumented for explainability by design. Japan brings standards-driven governance, including national guidance on generative AI in education and a revised nursing model core curriculum that supports outcomes-aligned blueprints and auditability [12-14]. We argue that combining Taiwan’s agility with Japan’s assurance can yield a credible regional reference model aligned with international guidance on trustworthy AI [1-6]. Although we write with nursing OSCEs in mind, the approach generalizes to other health professions.

## Aim, Key Messages, and Intended Audience

### Aim

This viewpoint proposes a practical governance and implementation blueprint for integrating generative AI into nursing OSCEs in ways that remain auditable, fair, and safe in high-stakes contexts.

### Key Messages

We argue that (1) AI functions in OSCEs should be decomposed into distinct roles (“5 AI roles”) to reduce uncontrolled automation; (2) each role must be paired with concrete governance artifacts (eg, machine-readable rubrics, persona cards, and versioned prompts and logs) and explicit human decision rights; (3) adoption should be phased (practice, mock OSCEs, and limited high-stakes stations) with predefined gates for reliability, validity, equity, and workload; and (4) cross-border learning is feasible if registry design defaults to the minimum necessary, deidentified data and ensures auditability.

### Intended Audience

The primary audience includes OSCE directors, nursing educators, simulation or standardized patient (SP) program leads, assessment committees, and institutional leaders responsible for educational quality and risk management. Secondary audiences include informatics teams, procurement, and legal and privacy offices.

## Why AI in OSCE Now—Promise and Pitfalls

OSCEs generate a wealth of multimodal evidence—verbal exchanges, checklists, global ratings, and timing data. Capturing that evidence consistently is labor intensive, and synthesizing it into timely feedback is even harder. Large language models (LLMs) and related tools can help standardize prompts and portrayals, extract evidence, and assemble individualized feedback. When done well, this can improve interrater agreement on observable behaviors, reduce rater workload, and shorten the feedback loop. Standardized, bounded, AI‑augmented SP (AI-SP) behaviors may also support equity by reducing unwanted variation in case portrayal.

However, the hazards are obvious. Unconstrained models can hallucinate, apply rubrics unevenly, and may amplify biases inherent in the data. Automated scoring can underrepresent constructs by overvaluing what is easy to detect and undervaluing tacit elements of professional judgment [11]. Overautomation can dull rater calibration and erode student trust. Model updates can drift silently, undermining comparability across cohorts. These risks argue for human-in-the-loop oversight, transparent logging, and governance grounded in accepted frameworks for trustworthy AI and risk management [1-6]. Nursing education must also track shifts in licensure and competency assessment—such as the Next Generation National Council Licensure Examination emphasis on clinical judgment [12]—and adhere to national guidance on responsible AI use [13,14].

## Evidence Snapshot: What Published Studies Suggest (and What Remains Uncertain)

Empirical studies on AI‑supported assessment and OSCE‑adjacent tasks suggest both promise and nontrivial limitations that matter in high‑stakes contexts. First, for structured written outputs scored against explicit rubrics, a peer‑reviewed JMIR Medical Education study compared ChatGPT (with GPT-3.5; OpenAI) scoring with SP scoring across 85 rubric elements in 168 first-year medical students’ SP-based history and physical notes (14,280 scoring opportunities) and reported a markedly lower incorrect scoring rate for ChatGPT (1%) than for SPs (7.2%) [15]. This supports the feasibility of LLM‑assisted scoring for checklist-appropriate elements under constrained prompts and clear rubrics while leaving open questions about generalizability across institutions, cases, and languages.

Second, a pilot randomized trial comparing an AI chatbot–based simulation with peer role-play for OSCE preparation reported complementary strengths (eg, autonomy and repeatable practice with structured feedback vs authenticity and an exam‑like environment), suggesting scenario‑dependent effects rather than uniform superiority [16]. Third, emerging transcript-based OSCE grading work applying LLMs to video-derived transcripts reports high alignment with human graders for selected rubric items (and includes failure analyses describing when reliability degrades) but also highlights sensitivity to prompting and risks of confident but incorrect rationales—reinforcing the need for evidence linking, uncertainty signaling, and human verification rather than autonomous scoring for high-stakes decisions [17].

Finally, the broader measurement literature on automated scoring emphasizes that fairness cannot be assumed; subgroup performance differences, construct underrepresentation, and drift require continuous monitoring with predefined indicators and remediation or rollback pathways [18]. Collectively, this evidence supports a phased adoption model in which AI remains advisory, each AI function is constrained by governance artifacts (machine-readable rubrics, persona cards, and versioned prompts), and scaling decisions are gated by reliability, validity, equity, and workload metrics. Related work has also proposed LLM-based approaches to evaluate SP simulations and has discussed whether LLMs can supplement (rather than replace) SP programs, reinforcing the need for bounded roles and governance in assessment contexts [19,20].

## A 5-AI-Role Model Across the OSCE Life Cycle

**Figure 1:**
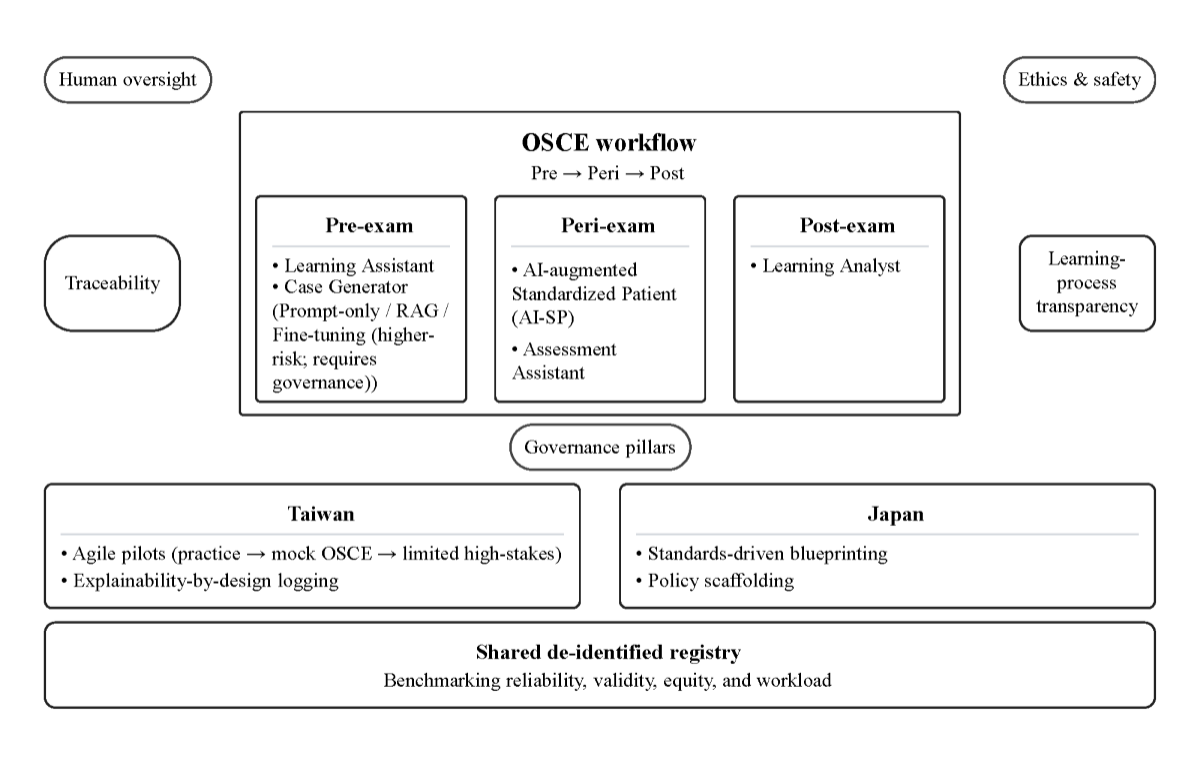
Conceptual framework for trustworthy artificial intelligence (AI)–augmented objective structured clinical examinations (OSCEs) in nursing education. The 5 AI roles—learning assistant, AI-augmented standardized patient (AI-SP), assessment assistant, case generator, and learning analyst—map to pre-OSCE, peri-OSCE, and post-OSCE workflows. Four governance pillars—human oversight, learning process transparency, ethics and safety, and traceability—surround the workflow. The Taiwan-Japan pathway layers agile pilots and explainability-by-design logging (Taiwan) with standards-driven blueprinting and policy scaffolding (Japan), feeding a shared, deidentified registry for benchmarking reliability, validity, equity, and workload. RAG: retrieval-augmented generation.

We propose a 5-AI-role model—learning assistant, AI-SP, assessment assistant, case generator, and learning analyst—mapped to pre-OSCE, peri-OSCE, and post-OSCE workflows. The intention is to augment, not replace, human expertise and make the assessment enterprise more transparent and improvable. Figure 1 sketches the overall architecture and its relationship to governance pillars and the Taiwan-Japan pathway, and Table 1 summarizes the 5 AI roles, governance artifacts, and human control points.

**Table 1:** Five artificial intelligence (AI) roles, governance artifacts, and human control points.

AI role	Primary function in OSCE^a^	Typical inputs	Typical outputs	Governance artifacts examples	Human control points	Key risks to monitor
Learning assistant	Practice support and formative feedback	Approved rubric, station objectives, and approved prompt set	Practice prompts and rubric-anchored feedback drafts	Prompt library, rubric versioning, and disclosure text	Allowed scope set by faculty and learner-contested outputs	Overscaffolding, misinformation, and privacy leakage
Case generator	Generate station or case variants within difficulty bands	Blueprint metadata, difficulty bands, and approved case library	Draft stations and localized variants	Difficulty bands, prohibited content rules, provenance notes, and faculty sign-off	Faculty review and approval before use	Unsafe or inaccurate content, cultural mismatch, and leakage of sensitive information
AI-augmented standardized patient	Standardized yet bounded interaction during stations	Persona card, response boundaries, and safety filters	Student-facing responses and interaction logs	Persona cards, safety or red-team checklist, model and version logs, and kill switch	Standardized patient trainer or faculty approval, with escalation route	Inconsistent portrayal, unsafe content, and bias in interaction
Assessment assistant	Evidence extraction and prescoring suggestions	Audio, video, or text and rubric items	Time-stamped evidence links and suggested checklist marks	Evidence-linking rules, uncertainty flags, and audit logs	Verified, edited, or rejected by a rater; final scoring done by a human	Construct underrepresentation, rater deskilling, and subgroup errors
Learning analyst	Post-OSCE feedback and monitoring dashboards	Scores, evidence logs, and rater actions	Individual feedback and reliability, equity, or workload metrics	Indicator definitions, access controls, and retention policy	Committee reviews trends and approves changes	Model drift, fairness gaps, and privacy risks from rich logs

^a^OSCE: objective structured clinical examination.

We refer back to Figure 1 as we outline the roles and the accompanying safeguards.

Before the exam, AI serves as a learning assistant and case generator. Institution-approved prompt sets and machine-readable rubrics seeded with blueprint metadata guide rehearsal of communication, reasoning, and procedural steps. Case generation produces variants that respect difficulty bands and cultural and linguistic localization; faculty always reviews outputs before use. Encoding rubrics and blueprints in structured formats enables consistent prompts and downstream alignment between station objectives, observable behaviors, and scoring criteria. This preparation supports explicit construct definitions and strengthens the validity argument that links observations to decisions [10,11].

For case generation, institutions can choose among three increasingly complex options: (1) constrained prompting using institution-approved templates, machine-readable rubrics, and difficulty bands (no model-weight updates); (2) retrieval-augmented generation grounded in an institution-approved local case library to improve factual consistency while preserving governance; and (3) fine-tuning, which should be considered only where there is a clear legal basis and robust controls (dataset provenance documentation; data minimization; deidentification where feasible; and predeployment evaluation against safety, bias, and leakage test suites). Regardless of approach, faculty review and sign-off remain mandatory before any generated case enters mock or high-stakes OSCE use.

During the exam, AI-SPs aim to improve standardization while allowing bounded adaptivity. Persona cards specify history, affect, cultural considerations, difficulty, and response boundaries, with model responses constrained to approved domains. Assessment assistants prescore specific behaviors or extract time-stamped evidence mapped to rubric items so that raters can verify, edit, or reject suggestions. Human assessors have final authority, and every AI suggestion comes with a rationale linked to auditable evidence, consistent with explainability by design [21].

After the exam, a learning analyst synthesizes performance into individualized, rubric-anchored feedback and cohort-level dashboards. Feedback emphasizes actionable suggestions and makes the evidentiary link to scores explicit. Cohort dashboards track blueprint coverage, rater effects, and subgroup performance differences to support continuous improvement and equity monitoring. Transparency artifacts—model cards and data sheets—document intended use, data sources, and limitations so that faculty can supervise with eyes open [22,23].

## Governance Pillars: Human Oversight, Transparency, Ethics and Safety, and Traceability

Trustworthy AI‑augmented OSCEs require governance that is rigorous yet workable. We summarize the 4 pillars that translate international guidance [1-6] into daily assessment practice.

First, human oversight ensures that AI remains advisory. Trained assessors make final judgments, using AI‑extracted evidence to inform rather than dictate scores. Calibration sessions can leverage AI‑curated exemplars to align interpretations of rubric items. Human‑in‑the‑loop routines minimize construct underrepresentation and preserve accountability central to programmatic assessment [9,11].

Second, learning process transparency is about showing how results were produced. For students, this means preserving prompts, drafts, and reflections. For assessments, it means keeping machine-readable blueprints, station rubrics, persona cards, and logs that capture who did what, when, and on which evidence. Explainability is judged by auditability rather than by opaque post hoc rationales [21-23].

Third, ethics and safety demand deidentification, data minimization, and guardrails against clinical misinformation, scope-of-practice drift, and inappropriate content. Safety checklists and red teaming apply to AI-SP prompts and assessment assistant outputs before deployment and after significant model updates. Procurement criteria and transparency artifacts create a baseline for safe integration, drawing on established digital technology assessment frameworks [24].

Fourth, traceability links choices to evidence across time. Standardized logs connect scores to time-stamped utterances or actions, raters, and model versions. This enables audits, drift detection, and equity monitoring. Where subgroup gaps arise, leaders adjust prompts, persona cards, or rubric anchors and repeat evaluation—treating fairness as an ongoing obligation in line with evidence of algorithmic disparities in health settings [25].

## Closed-Loop Monitoring and Improvement

During scoring, raters label each AI suggestion as accept, modify, or reject and optionally tag reasons (eg, “no supporting evidence,” “wrong rubric mapping,” “overstrict,” and “language or cultural mismatch”). These rater actions are logged alongside time-stamped evidence links and model or prompt versions and summarized in dashboards tracking error modes, drift signals, and subgroup differences. In high-stakes settings, optimization should preferentially occur through governance-layer updates (persona cards, prompt sets, rubric anchors, and extraction rules) because they are auditable and reversible; model retraining or fine-tuning is treated as a higher-risk intervention requiring explicit approval, revalidation, and a rollback plan.

## Complementary Strengths of Taiwan and Japan

Taiwan’s approach favors small-scope experiments that scale by evidence. Multidisciplinary task forces co-design cases, rubrics, and prompts; pilots start in practice environments to surface failure modes; mock OSCEs use A/B comparisons to quantify interrater agreement, time on task, and student workload. High-stakes use follows only after bias and error analyses are satisfactory. Explainability-by-design logs link every AI suggestion to specific utterances or events, and raters annotate accept, modify, or reject decisions. These habits align naturally with risk management frameworks that emphasize documentation, monitoring, and rollback capacity [4].

## Illustrative Taiwan-Informed Implementation Pattern (an Example)

In an agile pilot cycle, a small multidisciplinary task force (nursing faculty, simulation or SP trainers, informatics staff, and a privacy and ethics representative) co-designs (1) a machine-readable rubric, (2) an AI-SP persona card with response boundaries, and (3) a constrained prompt set for evidence extraction. The team conducts short red teaming sessions to intentionally trigger unsafe or biased outputs, revises prompts or personas accordingly and then runs a limited practice pilot. In a subsequent mock OSCE, stations are A/B tested with and without AI assistance to quantify rater time per station, interrater agreement, frequency of rater overrides (accept, modify, or reject), and student workload indicators. Only stations meeting predefined gates progress to limited high-stakes use.

Japan contributes policy scaffolding and institutional coherence. National guidance on generative AI articulates transparency, fairness, and responsible use that carry into higher education [13]. The 2024 nursing model core curriculum provides outcomes and assessment blueprints that support machine-readable rubrics, blueprint-to-station traceability, and integration with institutional research dashboards. Such scaffolding complements cautious, phased adoption and supports the accumulation of reliability and validity evidence consistent with OSCE best practice [7,8] and broader frameworks for trustworthy AI [5,6]. Alignment with the Next Generation National Council Licensure Examination’s focus on clinical judgment strengthens the case for explicit reasoning processes and feedback [12].

## Implementation Pathway and Practical Techniques

A staged pathway allows institutions to learn safely. Preparation includes encoding blueprints and rubrics in structured formats and creating AI-SP persona cards with response boundaries, affect, and cultural nuances. Faculty development favors minimal viable prompt sets for case generation, AI-SP control, and evidence extraction, paired with misuse-prevention checklists and short calibration workshops. Practice-environment pilots test persona stability, content safety, and evidence extraction quality. Mock OSCEs introduce AI‑assisted stations and compare conditions with and without AI support to quantify benefits and harms in context. Limited high-stakes rollout is reserved for stations where behavioral anchors are clear and AI‑extracted evidence is demonstrably reliable under human supervision.

Procurement and data governance underpin sustainability. Institutions should prefer tools that publish model cards and data sheets, expose application programming interfaces for log capture, support role-based access, and report subgroup performance [22,23]. Digital technology assessment criteria can be adapted for educational assessment to set minimum expectations for safety, security, interoperability, evidence, and transparency [24]. For cumulative improvement, traceability must be built into the system. Logs should record model versions, prompts, rater decisions, and time-stamped evidence to enable drift detection and recalibration.

Required manual interventions across the implementation pathway have 3 phases.

Phase 1 (practice setting) includes manually defining allowed prompts, approving rubrics and persona cards, running red-team tests, logging outputs, and reviewing failure cases on a fixed cadence (conducted by faculty).

Phase 2 (mock OSCE with A/B comparisons) includes manually assigning stations to either AI-assisted or standard conditions, conducting rater calibration, requiring raters to verify AI‑extracted evidence, and collecting student and rater workload measures.

Phase 3 (limited high‑stakes use) includes restricting deployment to stations with clear behavioral anchors, requiring a real‑time kill switch and escalation route, implementing an appeal pathway supported by traceable logs, and scaling only if predefined reliability, equity, and workload gates are met.

A brief worked example for the assessment assistant follows. The system highlights a time‑stamped utterance mapped to a rubric item; the rater verifies and clicks “accept.” For another item, the system suggests completion, but the rater observes no evidence and clicks “reject—no evidence.” Both actions are logged for audit and improvement.

## Evaluation Strategy: Reliability, Validity, Equity, and Workload

Evaluation should rely on a concise, theory-informed set of indicators. Reliability includes interrater agreement, internal consistency where appropriate, and stability of AI‑extracted evidence across sessions and subgroups. Validity evidence follows the inferences by Kane [11]—scoring, generalization, extrapolation, and decision—by linking rubric items to observable behaviors, checking generalization across variants, and examining decision consequences. Equity indicators track subgroup differences in scores, rater edits to AI suggestions, and the accessibility of feedback. Workload indicators include rater time per station, faculty preparation and calibration time, and student cognitive load. These indicators are reported at each deployment phase, with explicit gates for scaling. When subgroup gaps are detected, corrective actions target persona prompts, rubric anchors, or instructional supports and are followed by reevaluation, consistent with fairness-by-design practices [22,25].

## A Taiwan-Japan Road Map and Shared Registry

A joint road map can speed regional learning and comparability. Taiwan’s experience points to colocated educational engineering task forces, short A/B cycles, and audit-ready logging before high-stakes use. Japan’s policy scaffolding points to a stronger blueprint-to-rubric alignment, integration of AI-supported OSCE data with institutional research dashboards, and continuity of AI literacy and ethics across the curriculum. Together, partners can harmonize rubric vocabularies and metadata across Chinese, Japanese, and English; build a shared, deidentified registry of OSCE logs and outcome indicators; and run faculty exchanges and peer review to refine standards iteratively. The registry focuses on reliability, validity, equity, and workload; it records model versions and prompts to enable cross-site comparability and accompanies each dataset with transparency artifacts documenting intended use and known limitations [22-24]. This approach answers international calls for trustworthy AI, risk management, and auditable evidence pipelines [1-6].

## Position Within Assessment Theory and Practice

Our proposal aims to be compatible with established theory. The 5 AI roles address different parts of the evidentiary chain. The case generator and learning assistant support construct representation and learner preparation in line with blueprints, reducing construct underrepresentation [10,11]. AI-SP and assessment assistant emphasize consistent elicitation and capture of observable behaviors; human raters retain primacy in holistic judgments, which counters the tendency of automated signals to crowd out tacit expertise [9,11]. The learning analyst strengthens interpretation and use by providing timely, rubric-anchored feedback and surfacing patterns that inform teaching and remediation. In this way, AI can augment reliability and the feedback function of OSCEs while preserving the professional judgment central to programmatic assessment [9]. The approach also dovetails with licensure trends that foreground clinical judgment [12].

## Ethical and Legal Considerations

Ethics and legality must be handled in practical terms. Defaults should be data minimization and deidentification. Only the information needed for assessment, feedback, and improvement should be collected; access should be granted by role; and logs for audit periods should be set by policy. Content safety and cultural sensitivity should be tested before deployment with red teaming; inappropriate outputs trigger prompt revision or model replacement. Transparency with students and faculty about what AI is used for, what purpose it serves, and what evidence is retained is essential. When using third-party models, they require disclosures consistent with model cards and data sheets and insist on contractual guarantees for security and incident response [22-24]. Treat equity as continuous work, not a one-off checklist, acknowledging the empirical record of algorithmic disparities in health contexts [25]. National guidance on AI in education provides a baseline to adapt locally [13,14].

This viewpoint did not involve human participants or identifiable data; therefore, informed consent and institutional review board approval were not required.

## Cross-Border Legal and Data Governance (Taiwan’s Personal Data Protection Act and Japan’s Act on the Protection of Personal Information)

If OSCE artifacts (audio, video, transcripts, and rater notes) are used for monitoring or a shared registry, cross-border data governance becomes central. Taiwan and Japan both maintain comprehensive personal data regimes—Taiwan’s Personal Data Protection Act and Japan’s Act on the Protection of Personal Information—with practical implications for purpose limitation, data minimization, security safeguards, retention, and accountability. Because OSCE logs can include direct identifiers (faces, voices, and names) and indirect identifiers (rare scenarios, time stamps, and free-text notes), the shared registry concept should default to the minimum necessary, deidentified derivatives (eg, rubric-level scores, time on task, interrater statistics, and error-mode tags) and model and prompt version metadata. Where richer logs are needed for audit or research, additional safeguards are required (role-based access controls, retention limits, documented purposes, standardized deidentification or pseudonymization aligned with each jurisdiction, and contractual incident response). Where cross-border transfer constraints are substantial, federated analytics (local computation with only aggregate sharing) is a practical alternative.

## Limitations and Future Directions

This viewpoint is conceptual; it does not provide prospective evidence that AI‑augmented OSCEs improve reliability, validity, equity, or learning outcomes. Multisite pilots with predefined gates and common indicators are needed to test the framework in practice. Second, several technical elements are still evolving, including standardized approaches to explainability for conversational interactions, subgroup performance reporting, and robust drift monitoring and rollback across model updates. Third, the generalizability of persona prompts, rubric encodings, and machine-readable blueprints across languages, institutions, and specialties remains uncertain; controlled A/B studies and qualitative work with faculty and students are required to identify where localization or standardization is appropriate. Fourth, implementing audit-ready logging, bias monitoring, and multilingual harmonization may pose workload and infrastructure challenges—especially in resource-constrained programs—and could shift effort from teaching to tooling unless processes are simplified and supported. Fifth, data governance and procurement constraints (eg, contractual access to logs, model cards, and version histories) may limit transparency when third-party vendors are involved; practical templates and sector-level expectations are needed to raise a common floor. Finally, legal and regulatory contexts differ across jurisdictions, and alignment with licensure or accreditation requirements may lag; institutions should adapt governance to local requirements with stakeholder input and contribute results to shared registries so that evidence accumulates over time.

Even with these caveats, converging guidance on trustworthy AI, programmatic assessment, and transparency offers a feasible path from cautious pilots to safe, equitable scale [1-6,9,11,24-26]. A Taiwan-Japan collaboration can generate deidentified shared evidence, reduce duplicated effort, and help the region articulate standards that are contextually grounded yet internationally interoperable.

## Conclusions

AI can, with the right guardrails, make OSCEs more reliable, transparent, and feedback-rich while preserving human judgment. A 5-AI-role model integrated across pre-OSCE, peri-OSCE, and post-OSCE workflows—governed by human oversight, learning process transparency, ethics and safety, and traceability—provides a workable blueprint. Taiwan’s agile pilots and explainability-by-design logging, together with Japan’s policy scaffolding and blueprint-driven quality assurance, can form a credible Asia-Pacific reference model for trustworthy AI‑augmented OSCE in nursing education. A shared registry and harmonized vocabularies would accelerate collective learning. The pathway outlined here is actionable now, accords with assessment theory and international AI governance, and invites empirical testing through cross-border collaboration.

## Data Availability

No datasets were generated or analyzed in this study. Materials such as figure and table templates are available from the corresponding author on reasonable request.

## Abbreviations

AI: artificial intelligence
AI-SP: artificial intelligence–augmented standardized patient
LLM: large language model
OSCE: objective structured clinical examination
SP: standardized patient
